# T-Type Ca2+ Current Activity during Oocyte Growth and Maturation in the Ascidian *Styela plicata*

**DOI:** 10.1371/journal.pone.0054604

**Published:** 2013-01-22

**Authors:** Alessandra Gallo, Gian Luigi Russo, Elisabetta Tosti

**Affiliations:** 1 Animal Physiology and Evolution Laboratory, Stazione Zoologica Anton Dohrn, Napoli, Italy; 2 Institute of Food Sciences, National Research Council, Avellino, Italy; Universidad Nacional Autónoma de México, Mexico

## Abstract

Voltage-dependent calcium currents play a fundamental role during oocyte maturation, mostly L-type calcium currents, whereas T-type calcium currents are involved in sperm physiology and cell growth. In this paper, using an electrophysiological and pharmacological approach, we demonstrated, for the first time in oocytes, that T-type calcium currents are present with functional consequences on the plasma membrane of growing immature oocytes of the ascidian *Styela plicata.* We classified three subtypes of immature oocytes at the germinal vesicle stage on the basis of their size, morphology and accessory cellular structures. These stages were clearly associated with an increased activity of T-type calcium currents and hyperpolarization of the plasma membrane. We also observed that T-type calcium currents oscillate in the post-fertilization embryonic stages, with minimal amplitude of the currents in the zygote and maximal at 8-cell stage. In addition, chemical inhibition of T-type calcium currents, obtained by applying specific antagonists, induced a significant reduction in the rate of cleavage and absence of larval formation. We suggest that calcium entry via T-type calcium channels may act as a potential pacemaker in regulating cytosolic calcium involved in fertilization and early developmental events.

## Introduction

Oocyte maturation represents the last phase of oogenesis and consists of nuclear and cytoplasmic modifications [1]. Nuclear maturation is characterized by the meiotic process. In almost all species studied, the immature oocytes are arrested at first meiotic prophase (PI) which is characterized by a large nucleus, the germinal vesicle (GV). The PI arrest persists up to the time of the hormonal stimulus that resumes meiosis inducing the germinal vesicle breakdown (GVBD). This leads the oocyte to a second block occurring at different stages such as metaphase I (MI) in ascidians, bivalves and gastropods, and metaphase II (MII) in vertebrates and mammals. Finally, the second meiotic block is removed by the spermatozoon at fertilization, a cell interaction process where gametes recognize, bind and fuse to finally generate a new individual [2]–[5]. The cytoplasmic maturation is a less clear process characterized by morphological and functional changes that are necessary to support fertilization and the following developmental events [6]. In particular, cytoplasmic maturation is associated with a considerable increase in the oocyte size depending on the storage of foodstuffs and informational macromolecules, such as transcripts and proteins, modifications of plasma membrane and calcium (Ca^2+^) signalling [7]–[12].

Voltage-gated channels are located in the plasma membrane of many excitable and non-excitable cells, allowing ion currents to flow through the cell and giving rise to diverse physiological cellular processes. The role of ion currents in the gametes physiology has been well described in many animal species [7], [11], [13]–[16]. A pivotal role in gamete physiology is played by different types of voltage dependent Ca^2+^ currents; in particular, it has been shown that the high threshold L-type Ca^2+^ currents are either expressed in the immature oocytes and modulate oocyte growth, cytoplasmic maturation and early embryo development in a variety of organisms [9], [10], [17]–[20]. T-type Ca^2+^ channels are low voltage-gated channels that contribute to multiple physiological functions. By generating low-threshold Ca^2+^ currents, T-type Ca^2+^ channels influence action potential in neurons, impulse conduction in heart cells, myogenic tone in smooth muscle cells and hormone regulation in endocrine cells. In this respect, the importance of these channels in the physiopathology of human degenerative pathologies, such as cardiovascular diseases and cancer is recognized [21], [22].

In the gametes, the T-type Ca^2+^ currents are involved in the sperm physiology from spermatogenesis [23] to sperm activation by mediating the Ca^2+^ influx during the acrosome reaction process [24]–[26]. Limited evidence exists on the role of these currents in the oocyte. In the ascidian *Ciona intestinalis* a Ca^2+^ current, sharing some features of T-like, has been described in unfertilized oocytes [18], [27] and during spontaneous meiotic maturation in murine ovarian oocytes [28]. Apart from these few cases, T-type Ca^2+^ currents have never been reported to play a role during oocyte growth and maturation.

Ascidians are marine invertebrates commonly present worldwide representing a well-known experimental model in developmental studies [29], [30]. *Styela plicata* differs from other ascidian species since it lacks a clear reproductive apparatus; hence, many of the physiological processes related to its reproduction remain virtually unknown [31].

In order to characterize oocyte physiology and maturation in *Styela plicata*, we identified three different maturation and growth stages in oocytes collected from the ovary and described, for the first time, T-type Ca^2+^ current activity on the plasma membrane with an apparent functional role in the modulation of subsequent fertilization and early developmental events.

## Materials and Methods

If not otherwise stated, chemicals were purchased from Sigma-Aldrich (Milan, Italy).

### Animals and Gametes

Ascidians *Styela plicata* were collected from Gulf of Naples, a location that is not privately-owned nor protected in any way, according to the authorization of Marina Mercantile (DPR 1639/68, 09/19/1980 confirmed on 01/10/2000). The field studies did not involve endangered or protected species. All animal procedures were in compliance with the guidelines of the European Union (directive 609/86).

After collection, animals were maintained in tanks with running seawater at 18°C. Before use, they were anesthetized in ice and the ovary was dissected and oocytes collected with a Pasteur pipette and transferred to Petri dishes containing artificial sea water (ASW: 400 mM NaCl; 50 mM MgCl_2_; 10 mM KCl; 10 mM CaCl_2_; 10 mM HEPES, pH 8.2). Spermatozoa were collected with a fine Pasteur pipette from the spermduct and diluted in ASW before insemination.

Oocytes with an intact GV were selected according to Jeffery and Capco [32] and differentiated on the basis of oocyte size, cytoplasmic pigmentation and accessory cells morphology. The size of GV oocytes was evaluated, after denudation (see below), by measuring the diameter on a millimetre grid using an inverted microscope (Diaphot, Nikon Corporation, Tokyo, Japan). The surface area was calculated assuming a spherical shape.

### Electrophysiology and Pharmacology

In order to obtain nude plasma membrane for electrical recordings, the chorion and follicle cells of oocytes were removed by 0.03% (w/v) of Protease E from *Streptomyces griseus* in ASW. In *Styela plicata*, chorion and follicle cells are needed for fertilization; therefore, oocytes were dechorionated manually using steel needles after fertilization at the zygote stage, developed 30 min after the fertilization of fully grown oocytes. The nude oocytes and zygotes were washed twice in ASW, placed in Petri dishes coated with 1% (w/v) agar. Zygotes were used for whole-cell clamp recordings, or left to develop. In this case, at the appropriate stage (2, 4 and 8-cells embryos) were transferred to the recording chamber.

All experiments on nude oocytes and embryos were performed at room temperature (about 20°C) in a bathing solution containing 200 µl ASW. Ion currents were recorded in the whole-cell patch-clamp technique as previously described [10]. Briefly, patch pipettes were pulled using a Sutter P-87 (Sutter Instrument, Novato, CA, USA) with a tip of 1–2 µm in diameter showing a resistance of 3–5 megaOhms when filled with an intracellular-like solution (ICS: 200 mM K_2_SO_4_; 20 mM NaCl; 200 mM sucrose; 10 mM EGTA; 10 mM HEPES, pH adjusted to 7.5). Following the formation of a gigaseal, the membrane was ruptured by gentle suction obtaining the whole-cell voltage-clamp configuration. Currents and resting potential (RP) were measured using a List EPC-7 amplifier (HEKA Electronics, Cologne, Germany), filtered at 3 kHz and digitized with a Digidata 1322A under the control of pClamp9 software (Axon Instruments, Union City, CA, USA). To differentiate the high (L-type Ca^2+^) and low (T-type Ca^2+^) threshold currents, different holding voltages were used. Typically, a holding voltage of −30 mV for the L-type currents and −80 mV for the T-type Ca^2+^ currents were employed. However, similar to T-type Ca^2+^ channels, sodium (Na^+^) channels activate in the same negative range of membrane potentials.

Inward currents were elicited by depolarizing voltage steps from a holding potential of either −30 mV or −80 mV to +20 mV and +70 respectively in 10 mV increments to generate the voltage-dependent currents (I/V curves). To further characterize the ion currents observed at −80 mV, measurements were performed in: i. Na^+^-free ASW, prepared by replacing NaCl with 400 mM choline chloride and using KOH for pH adjustment; ii. Ca^2+^-free ASW, obtained substituting CaCl_2_ with 10 mM MgCl_2_ and adding EGTA 10 mM; iii. divalent-free (DW) ASW containing 460 mM NaCl; 10 mM KCl; 5 mM EGTA; 10 mM HEPES, pH 8.0; iv. 20 mM Ca^2+^ ASW. Moreover, pharmacological characterization of currents was performed by pre-incubating, for 30 minutes, samples in specific inhibitors of T-Type Ca^2+^ currents (25 µM NiCl_2_ and 1 µM mibefradil) and Na^+^ currents (0.1 µM tetrodotoxin; TTX). Control recordings were performed in ASW.

### Fertilization and Embryo Development

Aliquots of fully grown oocytes collected from the same ovary were fertilized adding about 10^6^ spermatozoa/ml in ASW (control) and ASW containing 25 µM NiCl_2_. One hour after fertilization, the number of 2-cell embryos was counted under the stereomicroscope and fertilization rate was calculated. Then embryos were left to develop to hatched larva in a culture chamber at 18°C.

Two-cell stage control embryos were also incubated in 25 µM NiCl_2_ and allowed to develop.

Vitality and morphology of hatched larvae were evaluated at the inverted microscope. In order to exclude a possible effect of NiCl_2_ on the spermatozoa, they were incubated for 30 minutes in the presence of the same concentration of NiCl_2_ and added to the dish containing fully grown oocytes.

### Statistical Analysis

The surface area of the oocytes and embryos was calculated assuming a spherical shape by using the formula 4πr^2^. Because cellular surface area change during growth ion current amplitudes were normalized and reported per mm^2^ of surface area to allow for an independent comparison.

The amplitude of ion currents is reported as mean ± standard error (S.E.) for the number of cells (n) in which whole-cell patch-clamp recordings were performed. Differences between peak values of electrical currents were analyzed with General Linear model (GLM) procedure of ANOVA [33]. In the case of values expressed as percentages, we proceeded to analyse data after arcsine transformation. Pair-wise comparison of the means were analyzed by the least significant difference (LSD) test.

## Results

### Oocytes Classification

**Figure 1:**
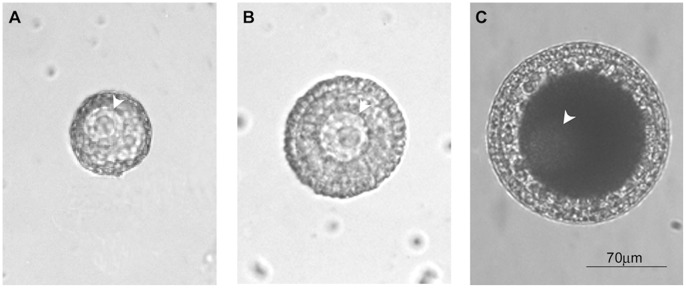
Representative images of the three GV stages in oocytes of *Styela plicata*. Panels A), B) and C) represent pre-vitellogenesis (stage A; GV-A), vitellogenesis (stage B; GV-B) and post-vitellogenesis (stage C; GV-C), respectively. The germinal vesicle is indicated (arrow head) in all stages reproduced.

Based on the size, oocyte pigmentation and changes in the morphology of accessory cells, we selected three subcategories of GV-containing oocytes as follow:

Stage A (GV-A), corresponding to pre-vitellogenic stage; oocytes were less or equal to 70 µm in diameter, with a transparent cytoplasm and a layer of flat follicle cells;

Stage B (GV-B), corresponding to vitellogenic stage; oocytes were 70–140 µm in diameter with a yellow cytoplasm and surrounded by a layer of columnar follicle cells;

Stage C (GV-C), corresponding to post-vitellogenic stage; oocytes were higher or equal to 140 µm in diameter, with a brown cytoplasm and surrounded by a layer of columnar follicle cells attached to a vitelline coat, with an innermost adherent layer of test cells (Figure 1).

### Ion Currents in GV Oocyte Stages

**Figure 2:**
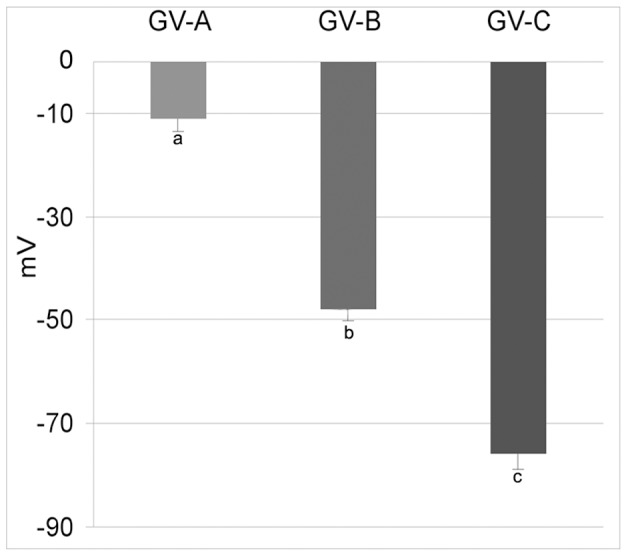
Membrane resting potential for different oocyte stages of *Styela plicata*. The resting potential (means ± S.E.) increased significantly through more negative values along the three stages (GV-A, GV-B and GV-C) described in legend of Figure 1 (a *vs* b *vs* c P<0.01).

**Figure 3:**
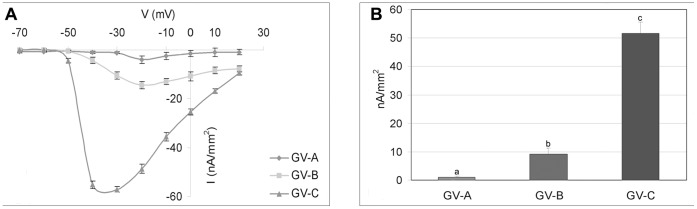
Electrophysiological characterization of ion currents during growth in oocytes of *Styela plicata*. A) Average current-voltage relationship (I/V curve) generated from depolarizing voltage steps between −70 and +20 mV from an holding potential of −80 mV. The current was maximally activated by voltage step to −20 mV in the GV-A and GV-B and to −30 mV in GV-C. All I/V curves are obtained by averaging the peak current for fifteen oocytes. Error bars indicate S.E. B) The maximum peak inward current (means ± S.E.) recorded at the activation step of −20 mV in GV-A and GV-B, while the value was −30 mV in GV-C (a *vs* b *vs* c P<0.01).

The resting membrane potential of the oocytes was −11±2.5 mV in the GV-A stage (n = 20). It increased significantly (P<0.01) during the oocytes growth as follows: −48±2.2 mV at GV-B stage (n = 25) and −76±2.8 mV at GV-C stage (n = 23) (Figure 2). The oocytes in the three stages were voltage-clamped at the holding potential of −30 mV. Applying the depolarizing voltage steps to test potential between −20 and +70 mV, we did not observe the inward component peaking at test potentials between 0 and +20 mV, commonly referred to as L-type Ca^2+^ currents. Depolarizing voltage pulses were then applied to the test potential between −70 and +20 mV, from a holding potential of −80 mV. Using this voltage-clamp protocol, it was possible to evoke an inward current in all the three stages. The activation threshold of the recorded inward current in GV-B and GV-C oocytes was −50 mV and the currents were maximally activated by voltage step to −20 mV in the GV-A and GV-B and to −30 mV in GV-C (Figure 3A). Activation and inactivation time constants at specific test voltage resulted as follows: 7.6±0.7 and 69.4±2.4 ms for GV-B at - 20 mV; 4.5±0.2 and 60.9±2.6 ms for GV-C at - 30 mV. The maximum peak inward currents in GV-A and GV-B at −20 mV were 3.9±0.2 nA/mm^2^ and 14.3±0.1 nA/mm^2^, respectively. At −30 mV, the value was of 57.1±0.6 nA/mm^2^ in GV-C (Figure 3B).

The selectivity to cations of recorded currents at GV-B and GV-C stages was examined since at these voltage steps either T-type Ca^2+^ and Na^+^ currents are activated. Oocytes at GV-B and GV-C stages, placed in either Ca^2+^-free ASW or DF-ASW, showed a significant reduction of the inward component respect to control (P<0.01). On the other hand, raising the external Ca^2+^ concentration to 20 mM resulted in a significant (P<0.01) increase in the amplitude of the inward currents (Table 1). In the absence of external sodium (Na^+^-free ASW), the inward currents did not differ from the control. Therefore, the characteristics and selectivity of the inward currents in GV-B and GV-C oocytes were similar to those of T-type Ca^2+^ currents.

**Table 1:** Ca^2+^ current amplitudes in control and treated GV-B and GV-C oocytes.

	GV-B*	GV-C*
**Control**	14.3±0.3	57.1±1.7
**Ca^2+^-free**	3.0±0.6	10.2±1.5
**DW-free**	2.5±0.5	5.8±1.6
**20 mM Ca^2+^**	19.55±0.4	71.3±1.4
**NiCl_2_**	6.7±0.3	28.4±1.1
**Mibefradil**	10.8±0.5	42.7±0.9
**TTX**	15.3±0.4	57.6±1.05
**Na^+^-free**	15.9±0.3	51.2±1.1

* n = 15.

Currents were recorded at the test potential of −20 mV in GV-B and −30 mV in GV-C.

**Figure 4:**
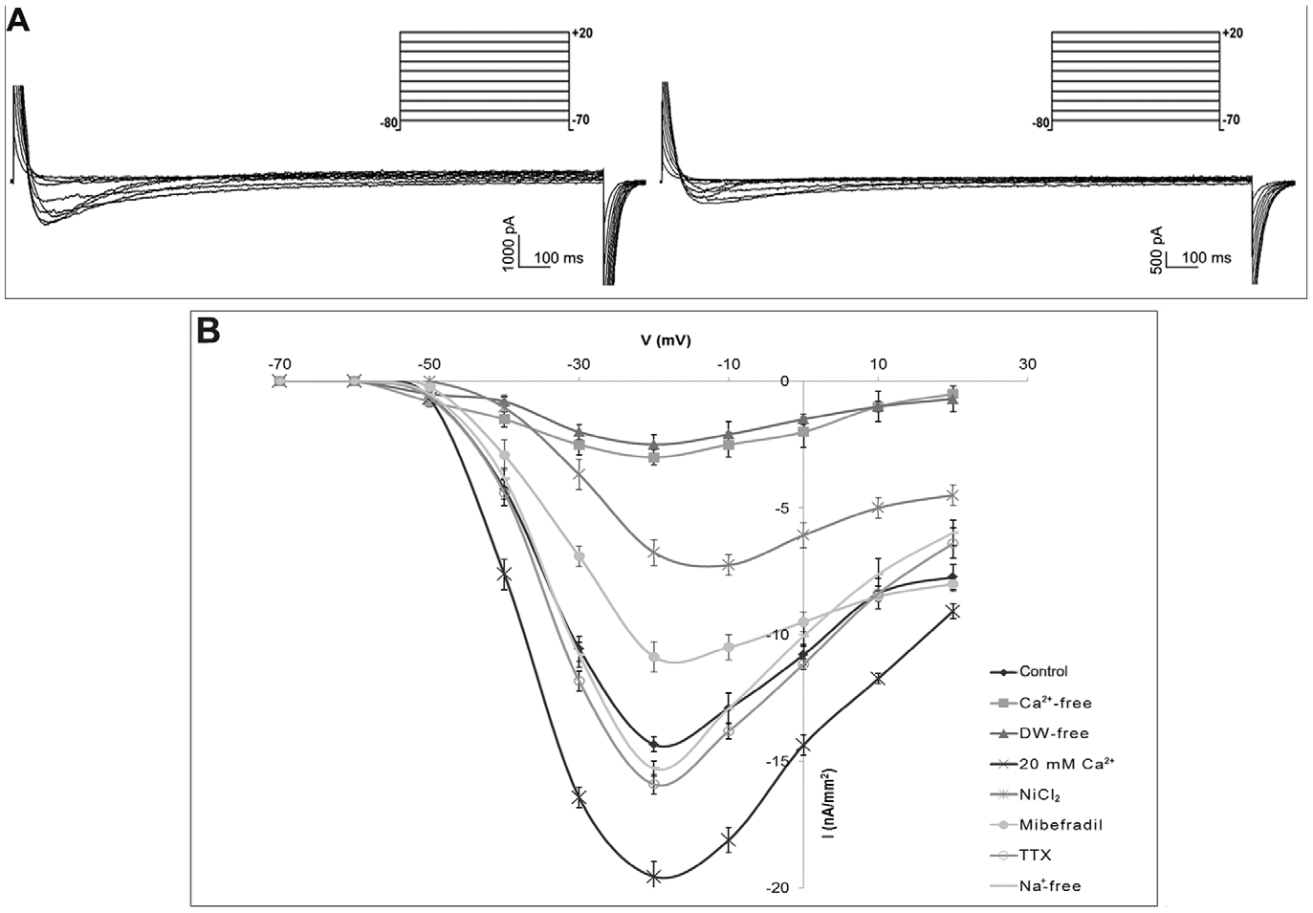
Pharmacological characterization of Ca^2+^ currents in GV-B oocyte of *Styela plicata*. A) Representative traces of voltage protocol and current records evoked by stepping membrane potential to voltages between −70 and +20 mV, in 10 mV increments, from a holding potential of −80 mV in control experiment (left) and after NiCl_2_ treatment (right). B) Average current-voltage relationship (I/V curve) showing the effects of modified ASW and pharmacological agents (NiCl_2_; TTX; mibefradil) on control currents. All I/V curves were obtained by averaging the peak current for twenty oocytes. Error bars indicate S.E.

**Figure 5:**
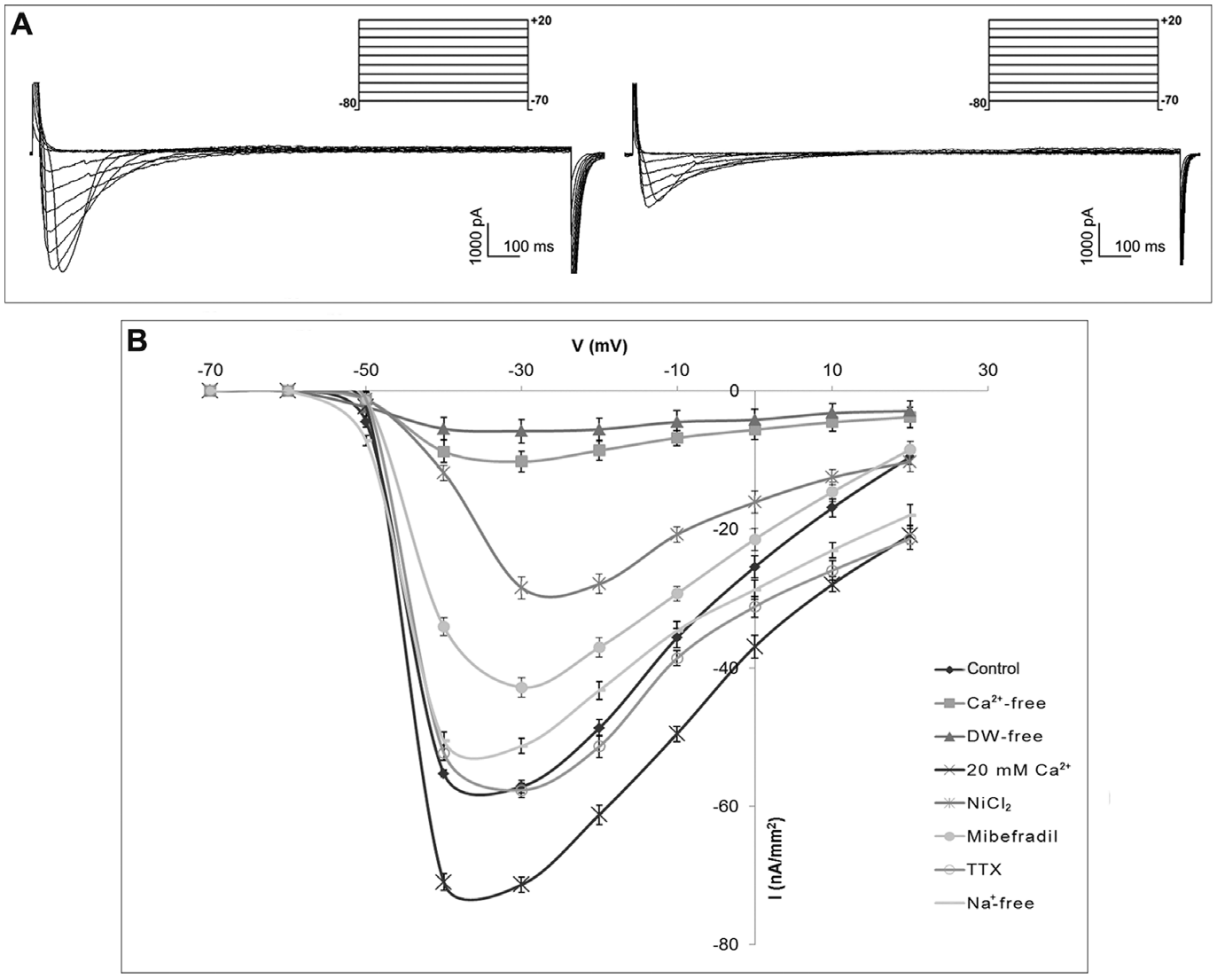
Pharmacological characterization of Ca^2+^ currents in GV-C oocyte of *Styela plicata*. A) Representative traces of voltage protocol and current records obtained by applying depolarizing voltage step between −70 mV and +20 mV, with subsequent 10 mV increments, from holding potentials of −80 mV in control experiment (left) and after NiCl_2_ treatment (right). B) Average current-voltage relationship (I/V curve) showing the effects of modified ASW and pharmacological agents (NiCl_2_; TTX; mibefradil) on control currents. All I/V curves are obtained by averaging the data from twenty oocytes. Error bars indicate S.E.

The inward currents in GV-B and GV-C were further characterized by examining their sensitivity to pharmacological agents (Table 1). The high sensitivity of T-type Ca^2+^ currents to be blocked by Ni^2+^ was selected as a specific signature of this channel. In addition, mibefradil was also employed as selective T-type Ca^2+^ channel blocker at submicromolar concentrations [34]. The inward currents were found to be sensitive to NiCl_2_ and mibefradil at 25 and 1 µM, respectively; in fact, it significantly decreased (P<0.01) in amplitude after pre-incubation of GV-B and GV-C oocytes in the presence both blockers for 30 min. The putative T-type Ca^2+^ currents were unaffected by 0.1 µM TTX, which specifically blocks Na^+^ currents (Figure 4 for GV-B and Figure 5 for GV-C).

### Ion Currents in Early Development Stages

**Figure 6:**
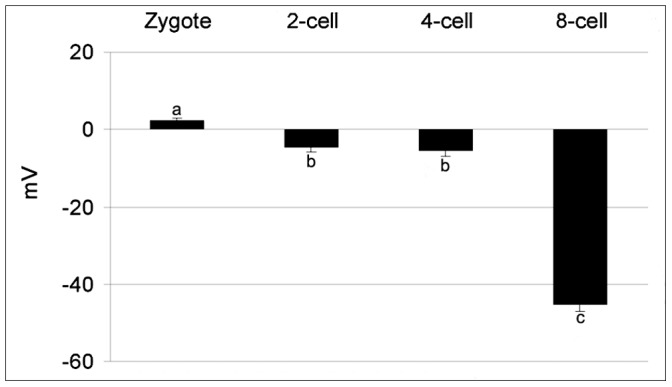
Membrane resting potential during embryo development in *Styela plicata*. The RP (means ± S.E.) shows positive values in the zygote. Negative values increased from 2-cell stage up to 8-cell stage (a *vs* b *vs* c P<0.01).

**Figure 7:**
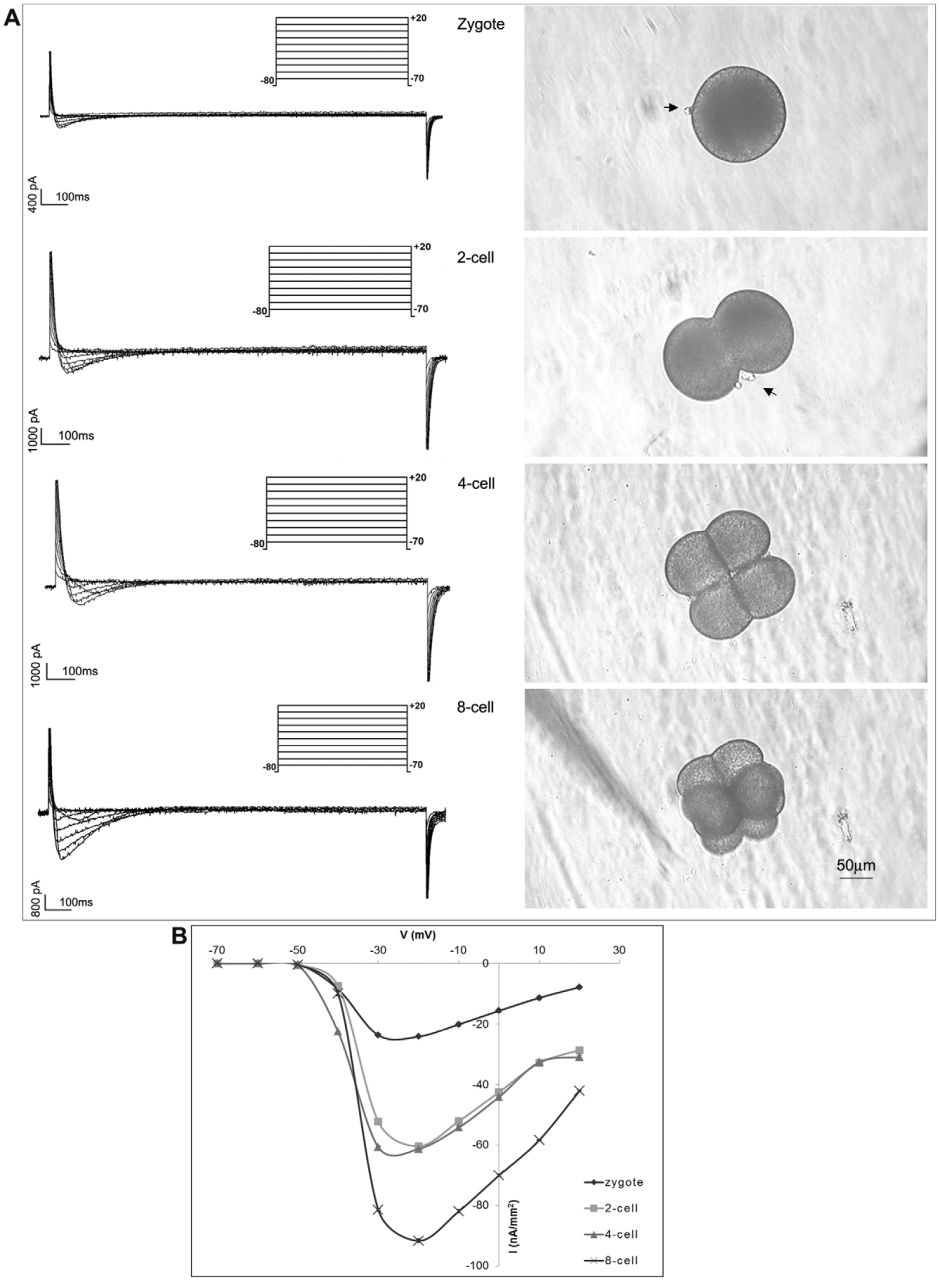
Electrophysiological characterization of Ca^2+^ currents during embryo development in *Styela plicata*. A) Representative traces of voltage protocol and current records obtained in response to voltage steps between −70 and +20 mV, in 10 mV increments, from a holding potential of −80 mV in the developmental stage reported (left). Images (right) at the light microscope of the stages considered. Arrows indicate the polar body. B) Average current-voltage relationship (I/V curve) showing that maximal currents generated at the step to −20 mV. All I/V curves are obtained by averaging the data from twenty considered embryo stages. Error bars indicate S.E.

Different embryo stages were held at −80 mV before application of voltage pulses between −70 and +20 mV. Fast transient inward currents were elicited during the depolarizing pulse. Typical current data recorded at each membrane potential and I/V relations are shown in Figure 6. In all stages analyzed, the threshold for activation was −50 mV and the currents were maximally activated at the test potential of −20 mV (Figure 7). An inward current was not elicited during the depolarizing pulses at the holding potential of −30 mV (data not shown).

In the evaluation of the maximal amplitude of the inward currents, we observed a significant increase during embryo development. At the zygote stages, the amplitude was 24.2±0.4 nA/mm^2^ (n = 9), significantly lower compared to the same current in GV-C oocytes (P<0.01). At 2-cell embryo stages, the currents increased to 60.4±0.2 nA/mm^2^ (n = 10; P<0.01) and did not differ in amplitude at 4-cells stage (61.3±0.2 nA/mm^2^, n = 10). In embryos at 8-cells, the inward currents increased again (91.7±0.3 nA/mm2, n = 9; P<0.01) (Figure 7). No amplitude differences were observed between blastomeres in all the considered stages.

Application of mibefradil resulted in reduction of the inward currents recorded in all stages considered (data not shown).

### *In vitro* Fertilization and Embryo Development

**Figure 8:**
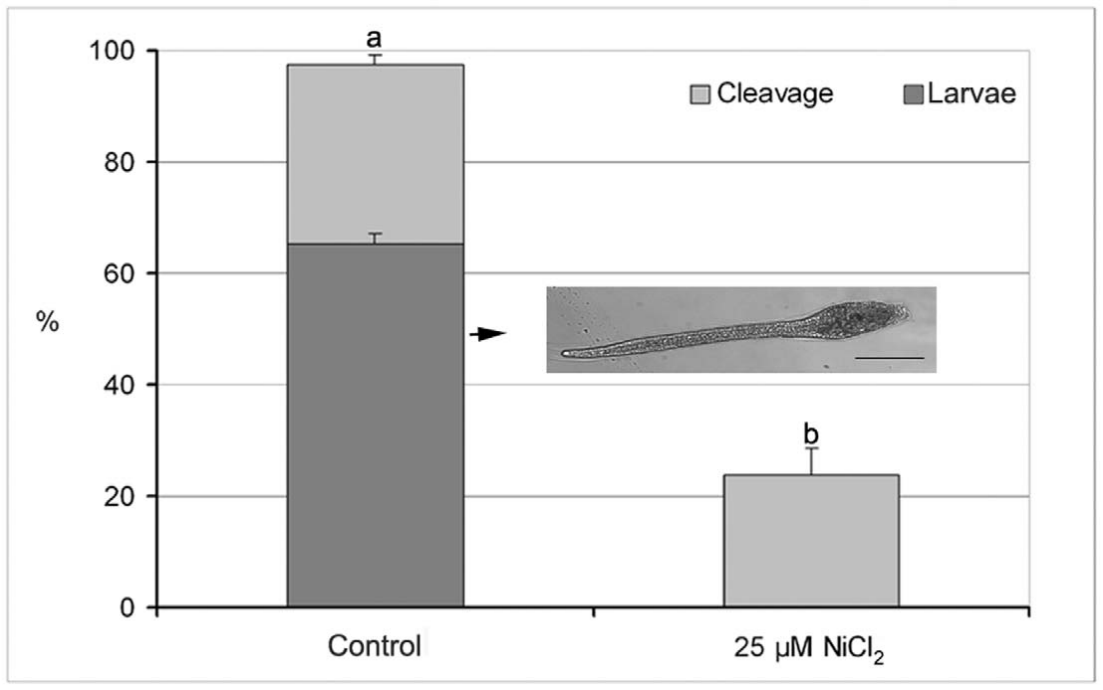
Effect of Ca^2+^ current inhibition on fertilization rate of *Styela plicata*. Light shading shows percentage of first cleavage (2-cell stage) of GV-C oocyte fertilized in ASW (control) and in NiCl_2_ (a *vs* b P<0.01). Dark shading shows percentage of 2-cell stage that reached the larval stage. Insert reports a normally developed *Styela plicata* larva 24 h after fertilization. Bar is 240 µm.

**Figure 9:**
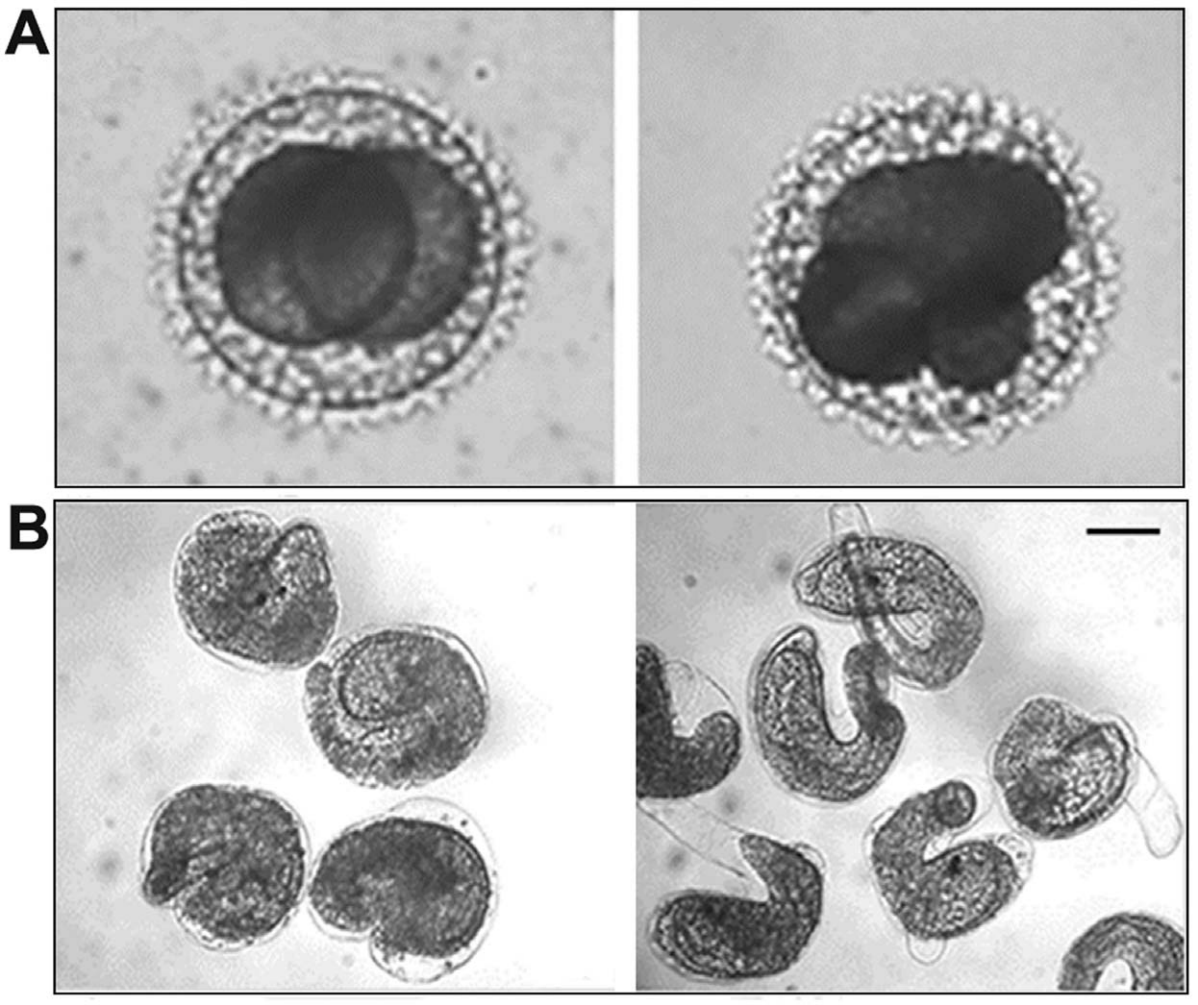
Effect of Ca^2+^ current inhibition on the *in vitro* fertilization and embryo development in *Styela plicata*. A) Slightly deformed embryos at 2-cell stage, developed from oocytes fertilized in ASW containing 25 µM NiCl_2_, arrested at an abnormal 8-cell stage; B) Abnormal hatched larvae developed from embryos transferred at 2-cell stage in ASW containing 25 µM NiCl_2_. Bar is 50 µm in A and 100 µm in B.

About 1 h after fertilization, *Styela plicata* oocytes undergo the first cleavage, with the subsequent early cell divisions occurring about 30 min thereafter. Larva hatched from the chorion 24 h post-fertilization. GV-C oocytes fertilized in 25 µM NiCl_2_ solution gave rise to lower percentage of cleavage (23.7±4.9%) respect to the control (97.4±1.9%; n = 15; P<0.01). After 24 h from fertilization, 65.2±1.8% of controls at the 2-cells stage reached hatched larval stage whereas in the presence of NiCl_2_, embryos arrested development at the 8-cell stage, showing abnormal morphology starting from the 2- cell stage (Figure 8; Figure 9A). When we transferred embryos at 2-cell stage, obtained by *in vitro* fertilization, into ASW containing 25 µM NiCl_2_ and left to develop, they gave rise to the same percentage of hatched larvae respect to the control, but all showing tail malformations (Figure 9B).

In experiments performed on spermatozoa incubated in NiCl_2_ solution and used to fertilize control oocytes, the fertilization and hatched larval rate did not significantly differ from the control (data not shown).

## Discussion

The role of Ca^2+^ as a second messenger involved in oogenesis and oocyte maturation is well described [7], [35]–[37]. The influence of Ca^2+^ entry through plasma membrane channels during oocyte maturation has been described only in few mammalian species [38]–[40]and mollusks [9], [11], [41]–[46]. In the majority of cases, the currents responsible for Ca^2+^ influx have been shown to be L-type Ca^2+^ currents [10], [17]–[19]. To date, nothing is known on the involvement of T-type Ca^2+^ currents in oocytes, while their function is normally associated to sperm physiology [25], [47], [48]. In this study, using electrophysiological and pharmacological approaches, we presented the first evidence on the existence of T-type voltage-dependent Ca^2+^ current activity on the plasma membrane of GV growing oocytes. We identified three stages of immature oocytes in *Styela plicata* on the basis of their size, morphology and accessory cellular structures (Figure 1) and characterized the electrical properties of their plasma membranes. The growth of GV-B and GV-C stages was clearly associated with an increased T-type Ca^2+^ current activity and membrane potential hyperpolarisation. In fact, the smallest oocytes (GV-A) exhibited a low Ca^2+^ current activity accompanied by the lowest resting potential value, which increased significantly during the following B and C stages.

The increase in Ca^2+^ current activity indicates that in *Styela plicata* oocytes, the initial stages of cytoplasmic maturation and growth progression may depend on a progressive Ca^2+^ surge via the plasma membrane. Intracellular Ca^2+^ release occurs at fertilization in all known species [7], [49]; in addition, modifications of Ca^2+^ stores during oocyte maturation may contribute to mediate Ca^2+^ release at fertilization [50], [51]. The highest density of plasma membrane Ca^2+^ currents in the larger GV-C stage indicates a plausible role of these currents in filling the internal Ca^2+^ stores that in the mature oocytes of ascidian *Ciona intestinalis* are responsible for the post-fertilization contraction of the zygote [52].

The completed maturation process represents a prelude to fertilization; fully grown oocytes are ready for the signal that resumes meiosis. In this respect, successful fertilization occurs only in presence of mature and competent oocytes [53]. In *Styela plicata*, fully grown GV oocytes are stored in the ovaries before spawning [54]. The literature reports many differences in oocyte maturation and spawning among ascidian species. In *Halocynthia roretzi* oocytes mature, just before spawning [55], in *Styela canopus* oocytes are spawned with intact GV which breaks down rapidly before fertilization [56], whereas *Styela gibbsii* oocytes are spawned after GVBD occurrence [57]. In *Styela plicata,* it was reported that oocytes are spawned with intact GV and that GVBD is triggered by the fertilization process [32]. In this study, we confirmed this observation, since the addition of spermatozoa to the GV-C stage oocytes triggered fertilization and embryo development up to larval stages, whereas the immature GV-A and B did not fertilize under the same conditions. Differently than other ascidian species, such as *Ciona intestinalis* and *Cnemidocarpa irene*, incubation of all immature stages in sea water was not followed by spontaneous maturation [10], [55], [57]. Based on these observations, we can hypothesize that the highest activity of Ca^2+^ currents recorded in GV-C stage may be necessary for the oocyte to respond to the unknown stimulus inducing resumption of the first meiotic block. The increase of RP also supports this hypothesis. Although the role of RP is not fully clarified, it has been found to vary during meiotic progression in some species [39], [40] and it appears to be associated with a “stand-by” status of the cell. It is possible that as soon as the plasma membrane receives the signal to grow, the ion exchange may induce the shift of RP through values closest to the physiological potential of the mature MI oocyte, supporting the metabolic activity necessary to prepare the plasma membrane for GVBD. In agreement with these results, we show here that plasma membrane potential significantly hyperpolarizes from stage A to C, reaching the most negative values in stage C, similarly to MI stage recorded in other ascidian species [24].

These data, along with the mitochondria pattern distribution recently demonstrated [58], suggest that the GV-C stage in *Styela* is considered the mature oocyte competent for fertilization.

The modifications in the plasma membrane potential have mostly been associated with the physiology of excitable tissues and related to cell cycle [59]. Several studies have shown that progression through the cell cycle is dependent upon transient increases in cytosolic Ca^2+^, since the inhibition of Ca^2+^ influx by the antagonists prevents cell cycle progression [60]. Regulation of some ion channels are dependent upon actin microfilaments [61]. During cleavage, the microfilaments reorganize to form the cleavage furrow to control changes in cell volume during mitosis. In *Ciona intestinalis*, perturbation of ion channels altered actin filaments organization and mitochondrial migration after contraction leading to a disturbance in cleavage formation [62] in agreement with the role of actin filaments in ion channel regulation [63]. These data are also consistent with the finding in somatic cells that a Ca^2+^ entry through T-type Ca^2+^ channels may be needed only at specific stages of the cell cycle for the control of cell growth and proliferation [64].

Functional expression of Ca^2+^ channels has been described during development in ascidians [10], [27]. In a previous study, we showed a T-type Ca^2+^ channel regulation by the cell cycle in the sea urchin embryo [65]. In the mouse oocytes, T-type Ca^2+^ currents increase after fertilization and decrease at the beginning of early development [66]; however, an involvement in the cell cycle regulation was subsequently shown in the early mouse embryo where the amplitudes of the T- type Ca^2+^currents change in a cell cycle-dependent manner being large in unfertilized oocytes and decreasing after fertilization throughout the first cell cycle and increasing again during late telophase [59]. In order to substantiate a possible role of T- type Ca^2+^currents in early development of *Styela*, we followed the pattern of current activity from zygote up to the 8- cell stage. The significant decline of either T-type Ca^2+^ currents and RP values occurring in the zygote up to 4-cell stage suggests a minor role for these currents in the signalling events related to the first embryonic mitotic cycle, whereas in either cases we observed a significant increase at the 8-cell stage without determining any specific differences among blastomeres. This finding appears in agreement with the critical role of the 8-cell stage embryo, where the segregation of cell lines initiates [31] and is consistent with data reported in *Ciona intestinalis* for L-type Ca^2+^ currents [10]. On the contrary, the lack of spatial distribution of currents among blastomeres at the 8-cell stage rules out a possible lineage-specific electrical diversity. The property and distribution of ion channels in embryos change during development [67]–[69]. In ascidians, it has been described an oscillation of Ca^2+^ currents that disappear at MI stage and reappear in the cells of muscular lineages [70].

Ca^2+^ influx through T-type Ca^2+^ channels may also be critical for cell cycle progression since their inhibition can prevent the proliferation of a variety of cell types including fibroblasts and endothelial cells [22]. In this paper, we demonstrated that the presence of functional T-type Ca^2+^ currents is critical for either fertilization and embryo development. In fact GV-C stage oocytes fertilized in NiCl_2_ arrest at 8-cell stage which, in ascidians, represents the fundamental stage for cell lines segregation and genomic activation [31]. T-type Ca^2+^ currents play also a pivotal role in development, since the treatment of 2-cell stage embryos with NiCl_2_ does not arrest embryos, but generates hatched larvae bearing serious morphological abnormalities of the tail, a key feature for larval metamorphosis in ascidians [31]. These data are also consistent with the impact of ion channels inhibition on late development in *Ciona intestinalis*, that coincides with the time of passage between maternal to genomic expression [10], [62].

### Conclusions

The present work shows the presence of functionally active T-type Ca^2+^ currents in immature growing oocytes of the ascidian *Styela plicata*. Several lines of evidence indicate that T-type Ca^2+^ currents play a role in growth regulation as suggested by their expression during embryo development and periods of rapid physiological and pathophysiological growth systems [71]. The significant increase of T-type Ca^2+^currents accompanied by the progressive hyperpolarization of the plasma membrane potential implies a peculiar role for these currents in regulating cytosolic Ca^2+^ during the cytoplasmic maturation and growth of *Styela* oocytes.

The absence of a clear GVBD and the fertilization occurring at the largest immature stage also suggest an important role of these currents in allowing Ca^2+^ entry that in turn triggers release of further intracellular Ca^2+^ from stores. The variation of T-type Ca^2+^ currents during development and the significant reduction of cleavage rate due to the inhibition of Ca^2+^ influx indicate that fertilization and embryo development are modulated by these currents.

Finally, we documented the difference between physiology of *Styela* oocytes and other ascidian species in which the Ca^2+^ entry at fertilization and post-fertilization are underlined by different channels types [10], [19]. These latter data further highlight the evolutionary variability of biological mechanisms that exist among the ascidian species [72].
